# Native American Pregnant and Postpartum People's Experiences of Discrimination During Perinatal Care: A Qualitative Study

**DOI:** 10.1111/1471-0528.70136

**Published:** 2026-01-07

**Authors:** Jennifer L. Murray, Heather Tanana, Torri D. Metz, Reham Perry, Elisabeth R. Kimball, Patrick Galyean, Susan Zickmund, Michael W. Varner, Michelle P. Debbink

**Affiliations:** ^1^ University of Utah Spencer Fox Eccles School of Medicine, University of Utah Salt Lake City Utah USA; ^2^ Department of Obstetrics & Gynecology University of Utah Spencer Fox Eccles School of Medicine Salt Lake City Utah USA; ^3^ University of Denver Sturm College of Law Denver Colorado USA; ^4^ New York University School of Global Public Health, NYC New York New York USA; ^5^ Qualitative Research Core, Division of Epidemiology, University of Utah Health Salt Lake City Utah USA; ^6^ Informatics, Decision‐Enhancement and Analytic Sciences Center, Veterans Affairs Salt Lake City Healthcare System Salt Lake City Utah USA

**Keywords:** American Indian/Alaska Native, Indigenous, maternal mortality, Native American, qualitative research, severe maternal morbidity, structural racism

## Abstract

**Objective:**

Indigenous pregnant and postpartum people are more likely to experience severe maternal morbidity (SMM) and mortality than non‐Indigenous groups. We sought to explore how community and societal factors, culture, and resilience impact Native American individuals' pregnancy experiences and might offer insights to address inequities in SMM.

**Design:**

Qualitative study.

**Setting:**

Virtual community focus group discussions.

**Population:**

Individuals who self‐identify as Native or Indigenous and gave birth in Utah within 5 years prior.

**Methods:**

We partnered with local Tribes and Tribal organisations to virtually conduct 3 focus groups of 3–5 participants and 1 individual interview. Open‐ended questions elicited participants' perceptions of their community and health system resources for perinatal care. Interviews were coded and analyses conducted to identify themes.

**Main Outcome Measures:**

Native individuals' perceptions of perinatal care focused on interactions with medical providers and the healthcare system.

**Results:**

12 participants enrolled. Participants reported harmful interactions that they believed were due to their Native American heritage. Four key themes emerged: participant experiences of discrimination, stereotyping, cultural dismissiveness, and explicit racism by their medical team. Participants suggested improving community support during and after pregnancy, holding cultural humility trainings for providers, and increasing representation of Native peoples in medicine.

**Conclusions:**

Native respondents endorsed several types of negative interactions with their medical providers during and after pregnancy that could affect their perinatal care. Efforts to reduce provider stigma and bias and improve cultural humility and awareness could improve these outcomes.

## 1 Introduction

Severe maternal morbidity (SMM) and mortality is a growing global health crisis that disproportionately impacts select racial and ethnic groups [1, 2]. Globally, Indigenous people experience disproportionately poorer maternal health outcomes than non‐Indigenous groups [3]. In the United States, Native American pregnant and postpartum individuals (Native mothers) experience maternal mortality rates up to 4‐fold higher than those of White individuals [4, 5]. There are relatively few studies focused on SMM among Native American pregnant people, partly due to insufficient Indigenous sample sizes and subsequent aggregation of racial and ethnic data in global, national, and state‐level data sources [3, 6]. Most current studies focus on medical risk factors for Indigenous SMM but often fail to consider social and structural contexts that might also contribute to this disparity as demonstrated in other marginalized populations [7, 8].

The 2010 World Health Organization conceptual framework for social determinants of health—the conditions in which people are born, grow, work, live, and age—identified racism as a structural and social determinant that drives health inequities by influencing social cohesion, capital, and the distribution of opportunities for health [9]. This framework has led to a better understanding of the impact of systemic racism and historical trauma on the health of Native Americans. However, few studies specifically evaluate maternal health—particularly among Native mothers residing in urban settings who may lack access to Indian Health Service facilities, which are primarily located on rural federal Indian reservations and provide government‐funded health care to Native people.

To address these knowledge gaps, we conducted an exploratory qualitative study to understand the broader socio‐structural factors that might contribute to the disproportionately high rate of SMM and mortality in Native American mothers. This study sought to elicit the range of participants' pregnancy and postpartum experiences, including supportive, neutral, and challenging aspects of care. Among the domains discussed, interactions with perinatal care providers and clinical settings emerged as a central and recurrent topic. Accordingly, this manuscript centers on participants' perceptions of their interactions with care providers—including physicians, midwives, clinicians, ultrasound technicians, and office staff—and the healthcare system in which those interactions occurred.

## 2 Methods

This was a qualitative study conducted between March 2022 and February 2023 and is reported in adherence to the Consolidated Criteria for Reporting Qualitative Research (COREQ) [10]. A completed COREQ checklist is provided in (Table S1). Eligible participants discussed their experiences during pregnancy and postpartum, describing the support they received and the challenges they faced. All participants provided written informed consent. The study was approved by the University of Utah Institutional Review Board.

### 2.1 Researcher Reflexivity and Positionality

The research team included both Indigenous and non‐Indigenous investigators with longstanding partnerships with Tribal and urban Indian communities across the Mountain West. While such relationships enhance participant and community trust, they can also introduce assumptions. Therefore, reflexive practices were incorporated throughout the study to minimise potential framing or confirmation bias [11]. These practices included community partner review of the interview guide, reflexive memo‐writing during coding, and regular team discussions to examine assumptions and ensure participant narratives guided analytic decisions. An author positionality statement is included in the Acknowledgements.

### 2.2 Patient and Public Involvement

We employed purposeful sampling to recruit participants in partnership with two Tribal health systems, Sacred Circle Healthcare (Confederated Tribes of the Goshute Reservation) and Four Points Health Clinic (Paiute Indian Tribe of Utah). We recruited through in‐clinic advertising as well as social media accounts of our partners and unaffiliated Facebook groups for Native Americans in Utah.

### 2.3 Setting and Sample

Participants were eligible if, at the time of the interview or focus group, they (a) self‐identified as Native American, American Indian, Alaska Native, and/or Indigenous, (b) lived in Utah, and (c) had given birth in Utah within the past 5 years. Individuals were ineligible if under 18 years of age or non‐English speaking, criteria that, to our knowledge, did not exclude individuals who were otherwise eligible. Participants were required to have access to a device (i.e., a cell phone, tablet, or laptop) that supports video conferencing and feel comfortable navigating a virtual focus group discussion. Individuals completed either an online or printed screener to indicate interest, provide socio‐demographic information, and determine eligibility. All focus group participants received a $75 gift card in appreciation for their time.

### 2.4 Data Collection

We used REDCap software to build and administer the participant screener. Demographic data were analysed in STATA‐18. All focus groups and the individual interview were conducted virtually by the Principal Investigator and senior author (MPD), an experienced clinician and qualitative researcher with formal training in interview and focus group facilitation. Select sessions were observed by an Indigenous member of the study team who served as the Tribal and community liaison (HT) and holds expertise in qualitative data collection. No other individuals joined these sessions. Prior to discussion, participants were made aware of the study's specific aims and the researchers' professional backgrounds and qualifications (MPD, HT).

The discussions were audio‐recorded and professionally transcribed, then analysed using the qualitative research software ATLAS.ti (version 23). The interview discussion guide was developed in collaboration with community partners. Open‐ended questions were designed to elicit the full range of participants' experiences surrounding perinatal care and were intentionally strengths‐based and solution‐focused to explore perceptions of community and health system resources, resilience, and opportunities for improvement. The guide prompted conversation in the following topic areas: personal definitions of family, community, and neighbourhood; available support systems through pregnancy, delivery, and postpartum; where you live and how it impacted your experience; community supports and challenges; medical provider support and challenges; experiences where you received care; discrimination within or outside of your community; and recommendations to improve Native mothers' experiences in your community. The formal discussion guide can be found in Table S2 of the supporting information.

### 2.5 Data Analysis

Qualitative analysis was conducted in collaboration with the University's Qualitative Research Core (ERK, PG, SZ). The analysts had no prior relationships with participating Tribal communities or involvement in data collection, providing analytic distance and helping to reduce potential interpretive bias. A codebook was developed through an inductive, open‐coding process as analysts reviewed the interview transcripts. To ensure reliability, two analysts (ERK, PG) independently coded all transcripts and met regularly to compare interpretations. The codebook was iteratively refined through these discussions until no new codes or insights relevant to the study emerged and thematic saturation was reached. Codes were then reviewed for extant patterns, and standard grounded theory was applied to synthesise salient themes. The analytic team (ERK, PG, SZ) met throughout this process to resolve discrepancies through consensus. Participants were invited to provide comments and feedback on the analysis and findings. In this manuscript, we report themes specifically related to participants' experiences with the healthcare system and medical care providers.

## 3 Results

### 3.1 Participant Recruitment and Eligibility

A total of 37 individuals submitted the eligibility screener, and 6 were excluded for the following reasons: not identifying as Native American (*n* = 2), not delivering in Utah during the 5‐year study period (*n* = 1), and currently residing outside of Utah (*n* = 3). Three additional people indicated discomfort navigating video conferencing, and the study team could not contact them to discuss accommodations. Of the 28 individuals who met all eligibility criteria, 19 were successfully contacted for participation and 12 completed the study (63.2%). 11 participated in 3 semi‐structured focus groups, and one completed an individual interview due to same‐day cancellations by two other group members.

### 3.2 Participant Socio‐Cultural Characteristics

Participants had an average age of 31.5 years (range 20–41) and represented 7 unique Tribal communities. The group was socioeconomically and culturally diverse with a range of education levels, household incomes, and religious backgrounds. One participant indicated mixed White heritage (8.3%), and most reported non‐Hispanic/Latino ethnicity (72.7%). Participants primarily lived in urban (50.0%) or suburban areas (33.33%) and had an average household size of 4.4 (range 3–7). Additional socio‐cultural and demographic characteristics are reported in Table 1.

**TABLE 1:** Socio‐cultural characteristics of participants (*n* = 12).

Demographic	*N* (%)
Age, *M* (SD)	31.5 (6.59)
Total annual household income	
<$10 000–$24 999	2 (16.67)
$25 000–$39 999	—
$40 000–$49 999	3 (25.00)
$50 000–$74 999	4 (33.33)
$75 000+	3 (25.00)
Religious affiliation	
Atheist	—
Christian	4 (33.33)
Traditional or Native American religion	4 (33.33)
Other (including agnostic, atheist)	4 (33.33)
Highest level of education	
Less than high school	—
High school, some college, trade, or associate's degree	6 (50.00)
Bachelor's degree	3 (25.00)
Master's degree	3 (25.00)
Doctorate degree	—
Residence^a^	
Rural or Unknown	2 (16.67)
Suburban	4 (33.33)
Urban	6 (50.00)
Household size, *M* (SD)	4.42 (1.24)

^a^ Rural: population not included within a suburban or urban area; typically have a population < 2500. Suburban: population between 2500 and 49 999. Urban: population of 50 000 or more.

On average, the interview and focus groups lasted 68 min, with the shortest 51 min and the longest 106 min. Participants discussed the challenges they faced with their medical providers at length, endorsing several harmful interactions and similar experiences of racism and discrimination. However, not all participants recounted negative experiences; some expressed immense gratitude for their providers and felt that they had a positive, collaborative relationship throughout pregnancy and postpartum. These relationships were described as those in which providers listened to participants' needs, elicited their relevant cultural practices, supported the use of pregnancy advocates such as Indigenous doulas, and helped them overcome prior pregnancy and birthing trauma. Unfortunately, these experiences were the minority. Four themes became evident throughout these discussions: participant perceptions of discrimination, stereotyping, cultural dismissiveness, and explicit racism during interactions with medical providers. Despite these difficult conversations, participants ended each session by offering solutions to better support and empower Native mothers in pregnancy and postpartum. These themes are defined and supported by illustrative quotes in Table 2.

**TABLE 2:** Definitions and illustrative quotes for each theme and participant recommendations for growth.

Theme 1: discrimination	
Definition	Experiences of discrimination during patients' time in pregnancy, delivery, and post‐birth. This can include discrimination based on ethnicity/colour or other discrimination, such as financial discrimination
#	*Quotes*
1	“I think there's a very huge discrimination thing happening to Native American women who are pregnant in the Salt Lake area. I've ignored it for as long as I could until I had a traumatic experience, and now I cannot ignore it.”
2	“When I consider all the reasons why the provider [a direct entry midwife] I hired would not help me during active labor and just abandoned me, broke the contract, I can't think of anything that makes sense other than discrimination. If my skin colour hadn't been the colour it is now, how would she have been? Would she have been a little more professional?”
3	“I don't know if it was because of my race. I really don't. But the luxury of being non‐Native is that you don't assume that.”
4	“I know for myself that there is always kind of just a nagging constant thought of, ‘Oh, what if I'm the unfortunate percentage of women, Native women, who have to deal with malpractice from my doctor?’”
5	“I didn't feel like the support was fully there, but I'm used to that, and I don't care. I don't expect it. As a Native woman, I don't expect that. And that may sound sad, but it's facts. But that's how I look out for myself.”
6	“But I think when you're in that lower‐income status, you're kind of used to not the best treatment. You're kind of used to subpar treatment. And I would say that it was like that with my being on Medicaid. But now that I'm in this different economic status, I can see the difference. I'm like, ‘Whoa, I didn't know I had options.’”
7	“I was on Medicaid with my first pregnancy. Again, survival period. But with this last pregnancy, I had [private?] insurance, and so I could see the difference in care.”
8	“I'm trying to think about WIC and just these different programs, and I felt like I wanted the pregnancy to be this amazing experience and the support, and I didn't get that. I just felt like I was this low‐income, not educated‐ I felt that. I felt that in my interactions with people.”

Theme 2: stereotyping	
Definition	Experiences of negative stereotyping during patients' time in pregnancy, delivery, and post‐birth. This can include stereotypes based on ethnicity/colour or others, such as cultural beliefs or substance use patterns
#	*Quotes*
1	“’We don't want to release it [opioid pain medication] to you because we have a high rate of Native Americans and morphine addiction.’ And so they were trying to take preventative measures and they didn't want to give her [participant's mother] the medication [for me] at all. I'm in pain in a hotel room and my mom was fighting for me, and that's when I'm‐ I think I read the stats there, like, ‘Oh, apparently, this is a thing.’ And they're blaming all Native Americans that apparently we're morphine addicts, and this is why we're not allowed to get pain meds when we are in pain, and I completely was like, ‘That's so racist.’ Had it been anybody else, I felt like that wouldn't [happen to them?].”
2	“I'm trying to be a good person. I'm trying to be sober. I'm trying to make my life better, but all these setbacks were awful. And I felt like I was just the worst parent ever. And when you go somewhere and you go, ‘Okay. I need help. All these things, I need help with,’ they're looking at you like, ‘Okay. You're Native American. You're an alcoholic’ … Here I am trying to make my life better, and I'm trying to make everything better for my kids, but it's like every step forward is a step back just because I look brown.”
3	“…the health services here in a community is kind of tough, too, for a Native woman because they always judge you by something….when you go you up there seeking for help, they're like, ‘Okay. So who's this? Where is she coming from? What does she need? Is she on drugs?’ or, ‘Is she on alcohol?’ or anything. And I hate that. I think that's kind of how Salt Lake City is. I don't know.”
4	“And there's just so much judgement when it comes to these babies and being pregnant and then delivering, even with all the cultural taboos.”

Theme 3: cultural dismissiveness	
Definition	Instances where providers were dismissive of participants' culture or what was important to them. This includes a lack of accommodations and understanding, or having cultural practices, beliefs, and taboos that are ignored or dismissed
#	*Quotes*
1	“I affiliate with Native American Church, which means that we participate in eating peyote, which is considered a hallucinogen with the federal government. And so I ate peyote while I was pregnant. I had [inaudible] ceremony while I was pregnant. But that's not ever something we could ever disclose.”
2	“And so there are just so many things we could never disclose because you know they're going to judge you, they're going to look at you a little sideways, and they're never going to understand. And that's something that I was taught a very young age is when it comes to people who are non‐Native, they're never going to understand. So save your breath and don't even explain yourself. Do what you need to do.”
3	“They actually took it [my placenta] and said that they needed to test it. And I had to fight with them to get it, but they wouldn't give it to us or release it to us because they said that the chemicals that they used would harm the environment…”
4	“It's not this intense smoke comes out… I really needed that connection, especially when I was hooked up [in the hospital] and wasn't able to go outside…. usually when I'm in a situation like that, I use my sage and I use my sweetgrass. And so we would ask, and they would just say no.”
5	“I just think that they should be more open to our beliefs and what's important to us with no questions asked.”

Theme 4: explicit racism	
Definition	Instances where participants experienced more overt and explicit forms of racism which they believed was due to their Native American heritage
#	*Quotes*
1	“And we always say, ‘Don't go to that doctor. Don't go to that doctor. He's really racist.’ I don't know if he's racist, prejudiced or what, but we just tell everybody not to go to him. ‘Go to these other doctors. Find someone that's going to work with you.’”
2	“After it was all said and done, she never came in and checked on me. So it was weird to me. And it just felt snide. I want to say this in a very tactful way. For me, it was some White women BS [nonsense], just snarky. And I just thought it was sad.”
3	“…just kind of making inappropriate comments, and it's always usually because they just lack the understanding, kind of making fun of maybe sometimes or just kind of, yeah, not really understanding…”
4	“Okay‐” I kind of have to educate them, be like, “Oh‐” kind of help them learn or kind of correct them a little bit so they don't keep doing that, so they won't disrespect others.”
5	“And being a Native woman speaking to White women and the way they talk to you, there's a certain amount of fight that has to come forward or advocating for myself, but I don't care because I watched it. I'm not going to let it happen to me and my kids again, what happened to my family.”
6	“I did [experience racism]. I even fought through it. I even went to the Tribal leaders for it. And when it comes‐ the reason why I say that is I've seen‐ for me, as a Native woman, I fight for every‐ anybody I see is a Native woman, you know? I agreed myself. This is who I am. I'm a Native from now‐ I'm so happy to see a Native woman.”
Opportunities for Growth: participant suggestions on how to improve support throughout the perinatal period	
#	*Quotes*
1	“Representation matters. Because when I had to go in for my D&C for my first miscarriage and when I was in the hospital at the birthing center for my first live birth, two women of colour made such a difference for me. Not because of the colour of their skin, but they treated me like a human, like their equal.”
2	“It would just be nice if they would push other Natives to be in there, get involved. If they're going to be going to school for … OB‐GYN or any midwife … I know I wanted to do something like that, and then I was kind of pushed to the side. And so I just went a different work career way, so. Because it is hard trying to fight for positions.”
3	“I don't know how that [racial discrimination] can be improved, except by calling them out saying, ‘This is the work that you want to do. And people are paying you to do the work that you say that you're so passionate about…”
4	“…if I was receiving information from a doctor or a nurse that had information or training on Native people, I would be so shocked and would be so interested, and that would mean a lot for me.”

### 3.3 Theme 1: Discrimination

Most participants shared stories in which they felt discriminated against by their medical team leading to inadequate or harmful care. Most believed these interactions were likely due to their Native American heritage and culture.

I think there's a very huge discrimination thing happening to Native American women who are pregnant in the Salt Lake area. I've ignored it for as long as I could until I had a traumatic experience, and now I cannot ignore it.

One individual could not be certain whether the mistreatment she experienced was due to her race, though it is something she always considers.

I don't know if it was because of my race. I really don't. But the luxury of being non‐Native is that you don't assume that.

Some participants have experienced discrimination in multiple medical settings and were unsurprised to see this occur during pregnancy and childbirth.

Others began to expect discrimination as an inevitable part of their perinatal care as they continued to have children.

I didn't feel like the support was fully there, but I'm used to that, and I don't care. I don't expect it. As a Native woman, I don't expect that. And that may sound sad, but it's facts. But that's how I look out for myself.

Several participants endorsed feelings of discrimination based on their financial status, including feeling deprioritized or receiving care they perceived as lower quality compared to wealthier patients. Participants believed there was bias against patients insured through Medicaid—the publicly funded US insurance program for low‐income individuals and families—due in part to its relatively low reimbursement rates. One individual believed that the quality of care in their second pregnancy improved because they had replaced Medicaid with commercial (private) insurance, which is typically obtained through an employer or purchased individually.

I was on Medicaid with my first pregnancy. Again, survival period. But with this last pregnancy, I had [private] insurance, and so I could see the difference in care.

Participants also endorsed feelings of judgement and discrimination for accessing other resources designed to assist low‐income families such as the USDA's Special Supplemental Nutrition Program for Women, Infants, and Children (WIC).

### 3.4 Theme 2: Stereotyping

Participants experienced racial stereotyping from their medical teams, such as the belief that all Native American individuals are drug‐seeking or have substance use disorders. They believe that this stereotype led to poor pain management and withholding of standard pain medications by their providers, especially during labor and immediately after giving birth. One participant felt that their medical team presumed she required substance and alcohol use treatment despite having no medical history of past use. In contrast, participants who did use substances during pregnancy felt that their providers saw them as a stereotype rather than an individual, despite disclosing this information to obtain additional support and create a stable future for their child

… Here I am trying to make my life better, and I'm trying to make everything better for my kids, but it's like every step forward is a step back just because I look brown.

### 3.5 Theme 3: Cultural Dismissiveness

Some mothers withheld information from providers about participating in cultural practices in fear of being berated or judged. This included the consumption of peyote and other traditionally sacred plants for ceremonial purposes during pregnancy or using cradleboards in lieu of car seats when travelling home from the hospital.

…And that's something that I was taught a very young age is when it comes to people who are non‐Native, they're never going to understand. So save your breath and don't even explain yourself. Do what you need to do.

Many Native American communities have postpartum traditions involving the placenta, umbilical cord, and the infant's meconium. Several participants wished to partake in these traditions but faced pushback when requesting these sacred items. Some wished to smudge—burn traditional plants and medicines—to bless their hospital rooms but were not allowed due to open‐flame policies. Medical staff did not offer alternative accommodations nor consult the hospital chaplain. Overall, participants felt frustrated and disappointed that their medical providers did not support them in practicing traditional customs, and they wished providers were more open to learning about cultures and customs that differ from their own.

I just think that they should be more open to our beliefs and what's important to us with no questions asked.

### 3.6 Theme 4: Explicit Racism

There were times that participants experienced more overt and explicit forms of racism, which they believed to be due to their Native American heritage and culture. These instances included providers and their medical teams using microaggressions, telling inappropriate jokes, verbally perpetuating stereotypes, and generally using harmful language when referencing Native peoples. In response, communities will often share stories, provide mutual support, and warn Native mothers so they can avoid harmful providers, hospitals, or clinics.

And we always say, ‘Don't go to that doctor. Don't go to that doctor. He's really racist.’

And it just felt snide. I want to say this in a very tactful way. For me, it was some White women BS [nonsense], just snarky. And I just thought it was sad.

When experiencing racism, some participants feel that it is their responsibility to correct White individuals' behaviour and educate them on historical trauma to prevent future harm to other Native peoples. Some mothers responded to experiences of racism by empowering themselves to fight back, advocate for their needs, and do the same for the Native women around them.

…for me, as a Native woman, I fight for every‐ anybody I see is a Native woman, you know? I agreed myself. This is who I am.

### 3.7 Opportunities for Growth

Given these challenges, participants suggested improving community support during and after pregnancy to better navigate the medical system, understand their rights, and decrease post‐partum depression. Participants also expressed the need for better representation of Native American peoples in the medical field and to make existing initiatives more inclusive.

Representation matters. Because when I had to go in for my D&C for my first miscarriage and when I was in the hospital at the birthing center for my first live birth, two women of color made such a difference for me. Not because of the color of their skin, but they treated me like a human, like their equal.

Finally, participants suggested Native American‐specific trainings for providers to improve cultural humility, acknowledge and challenge harmful behaviour, and decrease stigma surrounding cultural practices and customs.

…if I was receiving information from a doctor or a nurse that had information or training on Native people, I would be so shocked and would be so interested, and that would mean a lot for me.

## 4 Discussion

### 4.1 Main Findings

In this qualitative study of Native mothers' pregnancy experiences, participants reported widespread experiences of discrimination, stereotyping, cultural dismissiveness, and explicit racism from their medical providers and care team. To mitigate these challenges, participants recommended cultural humility trainings for providers, increasing the Native American medical workforce, and promoting community support and advocacy for Native mothers during pregnancy and postpartum. Finally, our findings generated several potential research topics, particularly surrounding the intersectionality of Native mothers' socioeconomic status, urban and rural residence, educational attainment, and utilisation of Tribal and non‐Tribal health systems and how this might impact their perinatal experiences.

### 4.2 Limitations and Strengths

Qualitative studies are not intended to be generalizable to a broad population, but to provide thematic insights that can drive future research or interventions. Nonetheless, study limitations include the small number of participants and narrow geographical recruitment. Acknowledging this, we achieved deep thematic saturation and successfully enrolled multiple participants with diverse Tribal identities. Our findings highlight cultural similarities rather than differences, but we recognize the heterogeneity between urban Native people and those living on Tribal lands, as well as within and between Tribal communities. Most of the represented Tribes originate from the Mountain West, indicating a future opportunity to capture Indigenous perinatal experiences across the globe. All participants also identified as members of the urban Indian community, which excludes the experiences of individuals currently living in rural or frontier areas. However, the urban Indian community comprises over 70% of all Native individuals in the US and remains significantly understudied, so this study offers a valuable contribution to the growing body of literature [12, 13]. All participants identified as women and a majority as heterosexual (91.7%). Therefore, these findings might differ for two‐spirit and LGBTQ+ individuals.

Our study also has several strengths. Foremost among these was a strong partnership with Utah Tribal governments and Native American community organisations. The study team has formed long‐term relationships with these community partners to build mutual trust, ensure reciprocity in research, and address the challenges most important to them and their communities. Maternal morbidity and mortality were identified as top priorities by several Tribal leaders, resulting in an authentic, community‐driven project. Furthermore, to our knowledge, this study is among the first to provide deep narrative insight into Native mothers' pregnancy experiences, which may lead to opportunities to close gaps in pregnancy outcomes. Though this study focuses on Native Americans in the US, Indigenous communities globally face higher rates of maternal mortality and many face discriminatory and marginalising circumstances. Therefore, insights gained from this study could inform efforts to partner with other Indigenous peoples to improve maternal health.

### 4.3 Interpretation

Despite disproportionately high SMM and mortality rates among Native mothers, the literature related to maternal health among Native individuals remains limited. Our findings begin to address these gaps and contribute to an understanding of Native mothers' interactions with healthcare systems and providers in predominantly urban settings. The lack of data in this space stems largely from the historic exclusion of Native American people from federal and state‐level datasets, racial misclassification, and data aggregation [14, 15]. The Urban Indian Health Institute (UIHI) has formally termed this phenomenon “data genocide,” and specifically calls out the invisibility of Native Americans in maternal health data. Not only is there a critical need for additional studies to fill this gap in knowledge, but also to move beyond the numbers and engage community‐driven work to foster meaningful change [16].

Our findings broadly align with other US and international studies assessing the experiences of pregnancy and childbirth broadly among marginalised groups, many of which call for solutions rooted in community [17, 18]. Vedam and colleagues worked with marginalised groups (including Canadian First Nations groups) to develop and validate community‐driven survey instruments to ascertain experiences of mistreatment, racism, and loss of autonomy in decision making during the perinatal period [19, 20]. They demonstrated higher mistreatment rates and loss of autonomy among Indigenous, Black, and other pregnant people of colour. In the Listening to Mothers in California study, mothers reported experiences of discrimination and bias by their medical teams during their hospital stay when they delivered their baby, which they believed was based on their race, ethnicity, socioeconomic status, linguistic background, and limited or lacking health coverage [21].

Focus group and individual interview studies with Black, Pacific Islander, Middle Eastern, and Latina mothers identified similar themes. Participants attributed physical and mental health conditions to racism, discrimination, and mistreatment in maternity care [18, 22, 23, 24]. In these studies, participants called for interventions that go beyond “band‐aid solutions,” noting that one‐off, asynchronous anti‐racism trainings are insufficient. Instead, they recommend holding community support groups, offering doulas, and amplifying the voices of those with lived experience to healthcare workers [22]. These recommendations align with our own participants' descriptions of positive and collaborative provider relationships, which emphasise respectful communication, culturally responsive support, and involvement of doulas and community advocates. The CEREMONY perinatal substance use program in Utah offers a practical example of how cultural humility and community healing can be operationalised in perinatal care by integrating cultural teachings, Indigenous peer support, and trauma‐informed provider training and simulation [25].

Together, these findings and our own suggest that experiences of racism and discrimination among marginalised pregnant and postpartum people have deep salience; this is important because racism leads to health consequences. In addition to creating mistrust of healthcare systems and providers, experiences of racism and discrimination across the lifespan could cause chronic psychological and physiological stress, leading to chronic health conditions such as hypertension, depression, substance use disorders, and diabetes [26, 27, 28]. When medical systems expose Indigenous peoples to racism and discrimination—whether implicit or explicit—during pregnancy and postpartum, it actively causes harm to patients rather than enhancing their health.

Our data highlight the importance of developing culturally responsive perinatal healthcare systems as critical next steps. Culturally rooted programs already exist in several Tribal communities, both formally and informally, which may offer insights for developing programs in urban and sub‐specialised maternal care. For example, Native women from the Wind River Indian Reservation in Wyoming built a peer‐led prenatal program leveraging kinship and natural social ties to provide culturally integrated education and emotional support to participants and families [29]. Additionally, Family Spirit, a home‐visiting program grounded in cultural traditions to support Native families, has been shown to improve maternal health, increase positive parenting practices, and improve child development in rural and urban settings [30, 31, 32]. These two programs, in addition to others, could serve as guides for the creation of perinatal programs that are community‐driven and culturally responsive. This is especially pertinent for Native mothers with complex obstetric histories or who do not reside on or have access to federally recognised Tribal lands and their health systems, where most culturally integrated programs currently exist.

Our study participants recommended provider trainings surrounding stigma, bias, and cultural humility to better prepare providers to best care for, prevent harm, and build trust with their Native American patients. Several institutions offer general stigma and bias trainings, but additional efforts are needed to fit the needs of this unique population. For example, the US Centers for Disease Control and Prevention (CDC)'s HEAR HER campaign offers educational materials for individuals, families, and providers to reduce maternal morbidity [33]. The CDC has developed specific materials in this campaign designed to provide the best care specifically for Native American mothers [34]. Finally, participants recommended increased Native American representation in medicine, which has been shown to improve maternal and child outcomes in other racial groups [35].

Box 1 provides examples of individual clinician actions that may respond to participants' concerns and recommendations. However, it's important to note that changes in clinician behaviour alone are necessary but insufficient to eliminate the causes of discrimination and racism in healthcare. Healthcare systems also bear responsibility for these harms, and meaningful progress requires changes to the structural factors that shape perinatal care delivery.

BOX 1 **Clinical Practice Points.**

- Proactively ask about and respectfully integrate cultural beliefs and practices related to pregnancy, birth, and the postpartum period whenever safe and feasible
- Provide relational care by scheduling patients with consistent providers and facilitating warm handovers when transitions or referrals are needed.
- Maintain an updated list of relevant community resources such as Indigenous doulas, community health workers, and local Native‐serving organisations.
- Engage in ongoing self‐reflection on cultural humility, recognising that all providers hold unconscious biases and have a responsibility to mitigate their impact on care.

## 5 Conclusions

Native American mothers experience discrimination, stereotyping, cultural dismissiveness, and racism during perinatal medical care. Our findings call for immediate and tangible efforts to reduce provider stigma and bias, and for the development of culturally integrated perinatal care models that leverage community support.

## Author Contributions

J.L.M. contributed to data interpretation and led manuscript writing. M.P.D., H.T., T.D.M., and M.W.V. originated the Voices of Native Mothers Project and contributed to all stages of study development, data collection, data interpretation, and manuscript writing. E.R.K., P.G., and S.Z. completed the qualitative data analysis. All authors revised the manuscript for critical content and approved the final version of the manuscript.

## Funding

This research reported in this publication was supported by: the March of Dimes and ABOG as part of the Reproductive Scientist Development Program; the University of Utah CARES grant; and the National Center for Advancing Translational Sciences of the National Institutes of Health under Award Number(s) UL1TR002538. The funders had no role in study design, data collection and analysis, decision to publish, or preparation of this manuscript. The content is solely the responsibility of the authors and does not necessarily represent the official views of the National Institutes of Health nor any other funder.

## Ethics Statement

This study was conducted in accordance with the University of Utah's Institutional Review Board (IRB #00142346; approved August 2021). All participants received written and oral information and provided written consent for participation in the study.

## Conflicts of Interest

M.P.D. received salary support from the March of Dimes and the American Board of Obstetrics and Gynecology as part of the Reproductive Scientist Development Program. M.P.D. serves on committees for the Society for Maternal‐Fetal Medicine and the Society for Reproductive Investigation, and as the Norman F. Gant ABOG Fellow at the National Academy of Medicine, all not‐for‐profit organisations. She did not receive remuneration for these duties. In the last 3 years, M.P.D. has received consultancy fees/honoraria from the Association of American Medical Colleges for the CHARGE initiative. These roles are not directly related to the content of this manuscript. Unrelated to this work, T.D.M. is the site PI for a Pfizer study of Paxlovid in pregnancy, a Pfizer study of an RSV vaccination in pregnancy, and a Moderna study of an RSV vaccination in pregnancy. She also receives royalties from UptoDate on topics related to the trial of labor after caesarean delivery. No authors of the manuscript have declared any conflicts of interest related to this work.

## Supporting information


**Table S1:** COREQ (COnsolidated criteria for REporting Qualitative research) checklist.

**Table S2:** Focus group discussion guide.

## Data Availability

The data that support the findings of this study are available on request from the corresponding author. The data are not publicly available due to privacy or ethical restrictions.
