# Supplementary material for: Native American Pregnant and Postpartum People's Experiences of Discrimination During Perinatal Care: A Qualitative Study
Source: BJOG. 2026 Jan 7;133(5):1026–36. doi: 10.1111/1471-0528.70136 (PMC12949996; doi:10.1111/1471-0528.70136)
Supplement: Supplementary file 1 — Table S1: COREQ (COnsolidated criteria for REporting Qualitative research) checklist. [file BJO-133-1026-s001.docx]

**Table S1.** COREQ (COnsolidated criteria for REporting Qualitative research) Checklist.

| **Topic** | | **Item No.** | **Guide Questions/Description** | **Reported on Page No.** |
| --- | --- | --- | --- | --- |
| **Domain 1: Research team and reflexivity** | |  | | |
| *Personal Characteristics* | | | | |
| Interviewer/ facilitator | | 1 | Which author/s conducted the interview or focus group? | 8 |
| Credentials | | 2 | What were the researcher’s credentials? E.g., PhD, MD | 1, 21 |
| Occupation | | 3 | What was their occupation at the time of the study? | 8, 21 |
| Gender | | 4 | Was the researcher male or female? | 21 |
| Experience and training | | 5 | What experience or training did the researcher have? | 8 |
| *Relationship with participants* | |  | | |
| Relationship established | | 6 | Was a relationship established prior to study commencement? | 6–7 |
| Participant knowledge of the interviewer | | 7 | What did the participants know about the researcher? e.g. personal goals, reasons for doing the research? | 8 |
| Interviewer characteristics | | 8 | What characteristics were reported about the interviewer/facilitator? e.g. Bias, assumptions, reasons and interests in the research topic | 6–7, 21 |
| **Domain 2: Study design** |  | | |  |
| *Theoretical framework* | | | |  |
| Methodological orientation and Theory | 9 | What methodological orientation was stated to underpin the study? e.g. grounded theory, discourse analysis, ethnography, phenomenology, content analysis | 9 |  |
| *Participant selection* | | | |  |
| Sampling | 10 | How were participants selected? e.g., purposive, convenience, consecutive, snowball | 7 |  |
| Method of approach | 11 | How were participants approached? e.g., face-to-face, telephone, mail, email | 7 |  |
| Sample size | 12 | How many participants were in the study? | 7 |  |
| Non-participation Setting | 13 | How many people refused to participate or dropped out? Reasons? | 7–8 |  |
| Setting of data collection | 14 | Where was the data collected? e.g., home, clinic, workplace | 7 |  |
| Presence of nonparticipants | 15 | Was anyone else present besides the participants and researchers? | 8 |  |
| Description of sample | 16 | What are the important characteristics of the sample? e.g. demographic data, date | 9, Table 1 |  |
| *Data collection* | | | |  |
| Interview guide | 17 | Were questions, prompts, and guides provided by the authors? Was it pilot tested? | 8, Table S1 |  |
| Repeat interviews | 18 | Were repeat interviews carried out? If yes, how many? | N/A |  |
| Audio/visual recording | 19 | Did the research use audio or visual recording to collect the data? | 8 |  |
| Field notes | 20 | Were field notes made during and/or after the interview or focus group? | 8 |  |
| Duration | 21 | What was the duration of the interviews or focus group? | 10 |  |
| Data saturation | 22 | Was data saturation discussed? | 9 |  |
| Transcripts returned | 23 | Were transcripts returned to participants for comment and/or correction? | N/A |  |
| **Domain 3: Analysis and findings** |  | | |  |
| *Data analysis* | | | |  |
| Number of data coders | 24 | How many data coders coded the data? | 9 |  |
| Description of the coding tree | 25 | Did the authors provide a description of the coding tree? | 8–9 |  |
| Derivation of themes | 26 | Were themes identified in advance or derived from the data? | 9 |  |
| Software | 27 | What software, if applicable, was used to manage the data? | 8 |  |
| Participant checking | 28 | Did participants provide feedback on the findings? | 9 |  |
| **Reporting** | | | |  |
| Quotations presented | 29 | Were participant quotations presented to illustrate the themes/findings? Was each quotation identified? e.g., participant number | 10–16, Table 1 |  |
| Data and findings consistent | 30 | Was there consistency between the data presented and the findings? | 9–16, Tables 1 and 2 |  |
| Clarity of major themes | 31 | Were major themes clearly presented in the findings? | 10, Table 1 |  |
| Clarity of minor themes | 32 | Is there a description of diverse cases or a discussion of minor themes? | 10–11 |  |

Developed from: Tong A, Sainsbury P, Craig J. Consolidated criteria for reporting qualitative research (COREQ): a 32-item checklist for interviews and focus groups. International Journal for Quality in Health Care. 2007. Volume 19, Number 6: pp. 349 – 357
