# Supplementary material for: Native American Pregnant and Postpartum People's Experiences of Discrimination During Perinatal Care: A Qualitative Study
Source: BJOG. 2026 Jan 7;133(5):1026–36. doi: 10.1111/1471-0528.70136 (PMC12949996; doi:10.1111/1471-0528.70136)
Supplement: Supplementary file 2 — Table S2: Focus group discussion guide. [file BJO-133-1026-s002.docx]

**Table S2.** Focus group discussion guide.

| **Questions** |
| --- |
| 1. Introduction: Name, when/where you last delivered, and what community means to you. |
| 1. What support systems were available to you during pregnancy, delivery, and/or after birth? |
| 1. Think about the place where you live. What does it look like? How does your living situation and neighborhood support pregnant women? How does it make pregnancy harder? |
| 1. What is your community doing well to support pregnant women? What could your community do better to support pregnant women? |
| 1. What has been your experience with doctors who provide care during pregnancy? 2. What has been your experience at the places where you received care (e.g. hospital, birth center, etc.)? |
| 1. During pregnancy, have you or someone you know experienced discrimination within or outside of your community? |
| 1. What top recommendations will improve pregnant women’s experiences in your neighborhood or community? |
